# Serum BDNF levels before and after the development of mood disorders: a case–control study in a population cohort

**DOI:** 10.1038/tp.2016.47

**Published:** 2016-04-12

**Authors:** K Ihara, H Yoshida, P B Jones, M Hashizume, Y Suzuki, H Ishijima, H K Kim, T Suzuki, M Hachisu

**Affiliations:** 1 Division of Public Health, Department of Social Medicine, Toho University Faculty of Medicine, Tokyo, Japan; 2 Research Team for Promoting Independence of the Elderly, Tokyo Metropolitan Institute of Gerontology, Tokyo, Japan; 3 Department of Psychiatry, University of Cambridge and Cambridgeshire & Peterborough NHS Foundation Trust, Cambridge, UK; 4 Department of Psychosomatic Medicine, Toho University Faculty of Medicine, Tokyo, Japan; 5 Department of Adult Mental Health, National Institute of Mental Health, National Center of Neurology and Psychiatry, Tokyo, Japan; 6 Faculty of Medical and Health Sciences, University of Auckland, Auckland, New Zealand; 7 Institute of Gerontology, J. F. Oberlin University, Tokyo, Japan; 8 Division of Clinical Pharmacy, Department of Pharmacotherapeutics, School of Pharmacy, Showa University, Tokyo, Japan

## Abstract

Serum levels of brain-derived neurotrophic factor (BDNF) are low in major depressive disorder (MDD), and were recently shown to decrease in chronic depression, but whether this is a trait or state marker of MDD remains unclear. We investigated whether serum BDNF levels decrease before or after the developments of MDD and other mood disorders through a case–control study nested in a cohort of 1276 women aged 75–84 years in 2008. Psychiatrists using the Structured Clinical Interview for DSM-IV identified incident cases of mood disorders at follow-up surveys in 2010 and 2012: 28 of MDDs, 39 of minor depressive disorders (minDDs) and 8 of minor depressive episodes with a history of major depressive episodes (minDEs with MDE history). A total of 106 representative non-depressed controls were also identified in the 2012 follow-up. We assayed BDNF levels in preserved sera of cases and controls at baseline and at follow-up. Serum BDNF levels at baseline in cases of MDD, minDD or minDE with MDE history were no lower than those in controls. The decrease in the serum BDNF level from baseline to follow-up was greater in cases of MDD or minDE with MDE history than in controls or cases of minDD. These results show that serum BDNF levels are not a trait marker of MDD in old women but appeared to be a state marker. The different changes in BDNF levels among diagnostic groups suggest that MDD has a pathophysiologic relation to minDE with MDE history, rather than to minDD.

## Introduction

Brain-derived neurotrophic factor (BDNF) has critical roles in neural proliferation, growth and survival, and in nearly all aspects of neural circuit function.^1^ It is prevalent in the limbic region and prefrontal cortex, which are key areas related to mood. Postmortem studies have shown that cerebral BDNF is associated with major depressive disorder (MDD)^2, 3, 4^ and may be decreased in patients with depression. Furthermore, BDNF is peripherally abundant, and its serum levels are low in patients with MDD^5, 6^ but can be increased with antidepressant therapy.^6, 7, 8^ Thus, BDNF is a possible biomarker of MDD.

Before clinical studies of BDNF, animal studies showed that BDNF expression in the hippocampus, frontal cortex and other regions of the brain is decreased by stress,^9, 10^ that the decrease of BDNF is reversed by antidepressants treatment^10^ and that BDNF has antidepressant-like effects.^11^ The understanding of intracellular signaling has been increased, such as by the finding that upregulation of cyclic adenosine monophosphate response element-binding protein is related to the increase of BDNF in mood-related regions of the brain.^12^

By combining the evidence from neurophysiological studies with evidence from studies of morphological alterations caused by stress in animal brains,^13, 14, 15^ and by associating the morphological alteration to the decreased volume of the hippocampus in patients with MDD,^16, 17^ Duman *et al.*^18^ proposed the neurotrophin hypothesis, in which BDNF is involved in the pathophysiology of depression. Subsequently, the hypothesis was further developed^19^ by evidence from the earlier studies of the postmortem cerebral and peripheral blood.^2, 3, 4, 5, 6, 7, 8^

The neurotrophin hypothesis is strongly supported by clinical evidence.^20, 21, 22, 23, 24, 25, 26, 27^ A large-scale meta-analysis of patients with MDD has shown low serum levels of BDNF.^28^ However, the hypothesis remains challenged by several issues.^29^ One issue is the causal relationship between lower serum levels of BDNF and the development of MDD:^19, 28, 29^ is serum BDNF a trait marker^30, 31, 32, 33, 34^ or a state marker^33, 35, 36^ of MDD? Although most evidence relies on cross-sectional data, Bus *et al.*^36^ provided strong longitudinal evidence for a state marker showing a decrease of serum BDNF in chronic MDD. However, they did not control the effect of a history of MDD and they did not show the decrease in incident MDD. Whether serum BDNF is a trait or state marker of incident MDD remains unclear. To investigate whether serum BDNF levels decease before or after the development of MDD and other mood disorders, we longitudinally examined serum BDNF levels in cases and controls of mood disorders derived from a population cohort in which we considered the effect of MDD history.

## Materials and Methods

This was a case–control study in a cohort from which serum was preserved. The original cohort comprised 1289 participants of a baseline survey in October and November 2008, which targeted 10 948 women, aged 75–84 years, in Itabashi Ward, Tokyo. After excluding 13 persons (11 persons with MDD and 2 persons with dementia) identified through a baseline psychiatric evaluation, we established a new cohort of 1276 subjects. Two follow-up surveys were performed in October 2010 and October 2012.

Each survey of the cohort was performed at the Tokyo Metropolitan Institute of Gerontology. Survey periods were 14, 12 and 9 days in 2008, 2010 and 2012, respectively. The survey consisted of physical and psychological examinations and questionnaires that included a self-administered depression scale^37^ referred to in Japan as the Depression Scale Basic Checklist (DSBC). The total score of the DSBC is 0–5 points and when 2 or more points is the cutoff for screening depression in the elderly. Subjects suspected with the DSBC in 2008, 2010 or 2012 to have depression were asked to undergo a psychiatric evaluation. We also asked subjects not suspected of having depression to undergo psychiatric evaluation. They were from two samples: a convenient sample^38^ chosen from participants in the 2010 follow-up and a consecutive one comprising participants on 3.5 days of all survey periods in the 2012 follow-up.

Psychiatric evaluation was performed within 2 months after each survey. Psychiatrists used the mood episodes (A) module and optional disorders (J3 and J4) module of the Structured Clinical Interview for DSM-IV (SCID)^39^ and identified mood disorders during the past 1 month and previous life. As the SCID requires psychiatrists to identify the onset time of disorders, we could identify incident cases of mood disorders that developed between the baseline survey and follow-up surveys, excluding cases that suffered from MDD at baseline. In addition to mood disorders according to the *Diagnostic and Statistical Manual of Mental Disorders*, fourth edition (DSM-IV),^40^ incident cases could include minor depressive disorder (minDD) according to the criteria sets for further study in DSM-IV.^41^ We also included depression that meets the criteria for minDD except that there had never been a major depressive episode (MDE) or a dysthymic disorder (referred to as ‘minor depressive episode (minDE)'), as minDE is often treated in the same way as minDD, without being distinguished from it. The SCID also enabled us to identify the status, including current, full remission, partial remission or under treatment, of the mood disorders at the surveys when blood was drawn to preserve samples of serum. The GRID–Hamilton Rating Scale for Depression (HAMD)^42^ was used to assess all cases of mood disorders identified with the SCID, and subjects who scored 6 or fewer points on the HAMD were ultimately judged to not have any mood disorder, except for those who were judged to be in remission or partial remission of a mood disorder. We excluded persons from cases if they had taken any antidepressant at baseline. Subjects who were found in the 2012 survey to have no previous or current mood disorder and to have no antidepressant use in their lives comprised a control group. Subjects with 23 points or less on the Mini-Mental State Examination^43^ were not included in either the mood disorder groups or the control group.

Blood samples were stored at −80 °C. Serum collected in 2012 was assayed with an enzyme-linked immunosorbent assay in February or March 2013. The serum stored since 2010 for cases of mood disorders found in 2010 and serum stored since 2008 for all cases and controls were assayed in February through April 2014. Serum BDNF levels were measured with the BDNF E_max_ ImmunoAssay System (Promega, Madison, WI, USA), according to the manufacturer's instructions. In short, 96-well microplates were coated with anti-BDNF monoclonal antibody and incubated overnight at 4 °C. The microplates were incubated in a blocking buffer for 1 h at room temperature. The samples, which were diluted 50 times with assay buffer, and BDNF standard were placed in the microplates at room temperature under conditions of horizontal shaking for 2 h, and were washed with a buffer containing Tris-buffered saline and Tween 20. The microplates were incubated with antihuman BDNF polyclonal antibody at room temperature for 2 h and washed with the buffer. The microplates were then incubated with anti-immunoglobulin Y antibody conjugated to horseradish peroxidase for 1 h at room temperature, washed with the buffer and incubated for 10 min adding with peroxidase substrate of tetramethylbenzidine solution to induce a color reaction. The reaction was stopped with 1 n HCl. The absorbance at 450 nm was measured with an automated microplate reader. All measurements were performed in duplicate. The BDNF standard curve was linear from 7.8 to 500 pg ml^−1^, and the detection limit was 10 pg ml^−1^.

Demographic and clinical characteristics at the baseline survey and at the follow-up survey, when the cases and controls were assessed by psychiatrists, were compared among the MDD, minDD, minDE with a history of MDE (excluding minDE with a history of dysthymic disorder) and control groups using the *χ*^2^-test and the one-way analyses of variance. Compared characteristics included potential confounders, which were found in previous epidemiologic studies^44, 45, 46^ and clinical studies.^47, 48^ The associations between these characteristics and serum BDNF levels were examined with the analyses of variance or the Spearman's correlation coefficient. Serum BDNF levels at baseline were compared between each of the three mood disorder groups and the control group by the analyses of variance with Dunnet multiple comparison tests considering possible confounders. To examine whether the changes of serum BDNF levels from the baseline survey to the follow-up survey differed among the four groups, BDNF levels at baseline and those at follow-up when the cases and controls were identified were entered as dependent variables together in a repeated mixed model with Scheffe multiple comparison tests. Mixed analyses allowed us to use all available values without effects of missing values.^49^ We performed all analyses with the software program SPSS version 18.0 (SPSS, Chicago, IL, USA) and SAS version 9.2 (SAS Institute, Cary, NC, USA).

The study was approved by the ethics committee of the Toho University Faculty of Medicine (registration number 25106). Written informed consent was obtained at the baseline survey and at the follow-up surveys.

## Results

The number of participants in the follow-up surveys was 729 in 2010 and 570 in 2012 (Table 1 and Supplementary Figures 1 and 2). Of the persons who had been screened as positive with the DSBC, 111 had participated in the psychiatrists' evaluation in 2010 or 2012. Of the persons who had been screened as negative, 40 from a convenient sample in 2010 and 122 from a consecutive sample of 162 persons in 2012 had participated. Among the total of 273 participants, psychiatrists identified 80 cases of mood disorders developed during the 4-year follow-up period. Of the 9 persons who had minDE, only 1 person, who had a history of dysthymic disorder, was excluded from the analyses. Finally, incident cases were combined into three diagnostic groups: a MDD group of 28 persons; a minDD group of 39 persons; and a minDE with a history of MDE (referred to as ‘minDE with MDE history') group of 8 persons. These persons, except for 2 persons with MDD who were being treated during follow-up, had not received any antidepressant during the 4-year follow-up period. Among them, 10 persons in the MDD group, 37 in the minDD group and 8 in the minDE with MDE history group were in a current episode. The control group consisted of 106 persons.

Table 2 shows demographic and clinical characteristics from the follow-up surveys in 2010 or 2012, when cases and controls were identified. There were significant associations between diagnostic groups and (a) sports and exercise activity, (b) the time difference between the baseline survey and the onset of current episodes and (c) storage duration of sera between blood draws and BDNF assays. In 5 of the 6 persons of the MDD group with a history of MDE, the first episode had developed after an age of 65 years. Among the characteristics listed in Table 2 (details not shown in the table), a history of MDE, smoking and alcohol drinking were related to serum BDNF levels; mean BDNF levels were higher in persons without a history of MDE (11.5 (s.d.=4.4) ng ml^−1^) than in persons with a history (6.9 (4.0), *P*=0.036), in smokers (16.7 (8.8)) than in nonsmokers (11.0 (6.2), *P*=0.030) and in persons who drank 4 or fewer days per week (11.5 (6.3)) than in persons who drank 5 or more days (6.9 (5.1), *P*=0.020). The storage duration of sera was correlated with BDNF levels; a person with longer storage duration had a higher BDNF level (*ρ*=0.181, *P*=0.021).

Characteristics at baseline, except for the storage duration of sera, did not differ among the four diagnostic groups (Table 3). Among these characteristics (details not shown in Table 3), alcohol drinking was related to serum BDNF levels (11.6 (s.d.=6.4) ng ml^−1^ for persons who drank 4 days or less per week and 7.3 (4.5) for 5 days or more, *P*=0.021). Mean BDNF levels differed between whether a history of MDE was present (14.8 (3.5)) or absent (12.9 (3.3), *P*=0.044). The serum BDNF level at baseline did not differ between the mood disorder groups and the control group; after the effect of the storage duration of sera, alcohol drinking and a history of MDE and the timing of blood drawing (morning or afternoon) were controlled.

**Figure 1:**
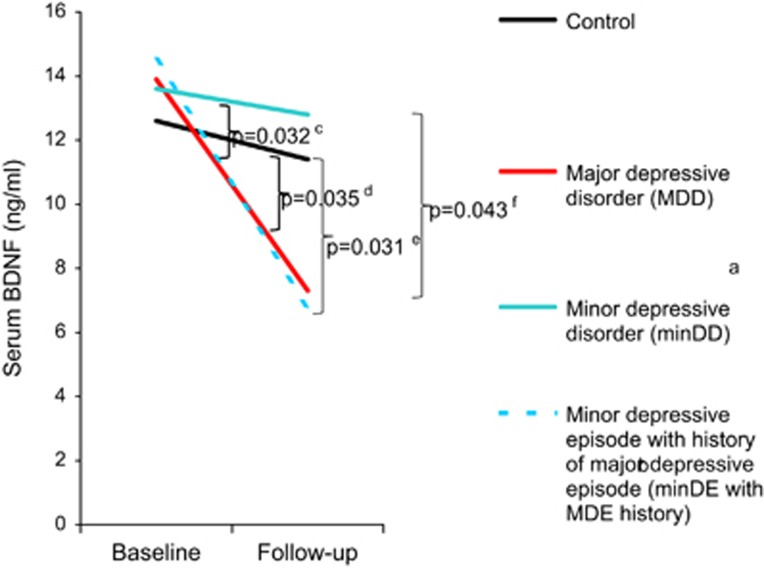
Changes of serum brain-derived neurotrophic factor (BDNF) levels from baseline to follow-up among the three incident mood disorder groups and the control group. *P*-values were calculated with Scheffe multiple comparisons of interactions between time and each of the diagnostic groups in a repeated mixed model. ^a^Minor depressive disorder (minDD) was diagnosed according to the criteria sets for further study in *Diagnostic and Statistical Manual of Mental Disorders*, fourth edition.^41^ ^b^Minor depressive episode (minDE) was defined as depression that meets the criteria for minDD except that there had never been a major depressive episode (MDE) or a dysthymic disorder. ^c^Between minDE with MDE history group and minDD group. ^d^Between major depressive disorder (MDD) group and control group. ^e^Between minDE with MDE history group and control group. ^f^Between MDD and minDD groups.

**Figure 2:**
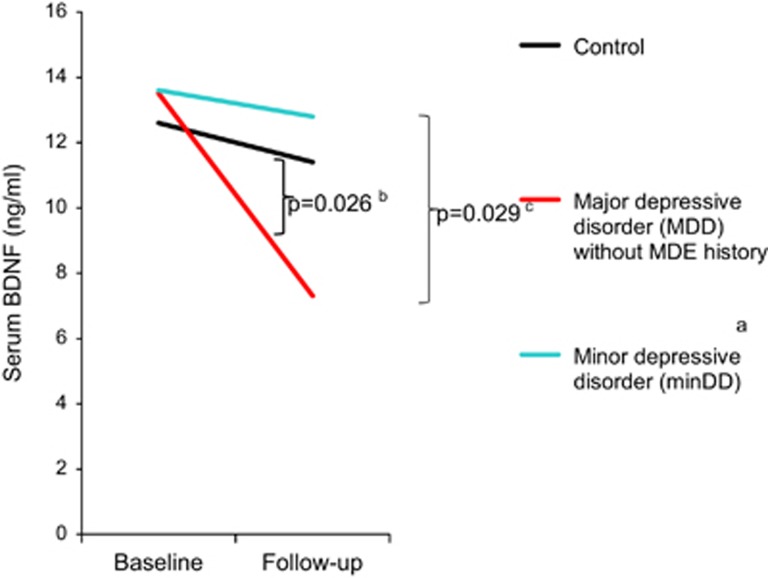
Changes of serum brain-derived neurotrophic factor (BDNF) levels from baseline to follow-up among the two incident mood disorder groups and the control group when excluding persons with major depressive episode (MDE) history. *P*-values were calculated with Scheffe multiple comparisons of interactions between time and each of the diagnostic groups in a repeated mixed model. ^a^Minor depressive disorder (minDD) was diagnosed according to the criteria sets for further study in *Diagnostic and Statistical Manual of Mental Disorders*, fourth edition.^41^ ^b^Between major depressive disorder (MDD) group and control group. ^c^Between MDD and minDD groups.

Mean serum BDNF levels at follow-up in cases that had not been treated with any antidepressant but had fulfilled the diagnostic criteria of current episodes for each mood disorder were 7.3 (s.d.=3.7) ng ml^−1^ for MDD, 12.8 (5.9) for minDD and 6.8 (4.2) for minDE with MDE history (Figure 1). Mean serum BDNF levels in other groups at follow-up were 11.4 (6.6) for control, 13.7 (3.7) for MDD in partial remission, 13.8 (7.8) for MDD in full remission and 11.3 (12.0) for under treatment. Entered into a repeated mixed model for dependent variables were as follows: the BDNF values at follow-up of controls and persons with current depression episodes but not in full remission, partial remission or under treatment; and all available values of BDNF at baseline. Entered into the model as confounders were the following: alcohol drinking, timing of blood drawing and storage duration of sera at baseline; the habits of smoking and drinking alcohol, activity in sports or exercise, timing of blood drawing and storage duration of sera at follow-up; time difference between baseline survey and follow-up survey; the time difference between the baseline survey and the onset of current episode; the history of MDE; and age at psychiatrists' assessments. After the effects of the confounders were controlled, the interaction between time and diagnostic groups was significant in the model (*P*=0.0009). It is only smoking at follow-up that was found to have a significant fixed effect among the confounders in the model (*P*=0.030). Multiple comparisons of interactions between time and each pair of the diagnostic groups showed that BDNF levels decreased more over time in the MDD group and the minDE with MDE history group than in the control group or minDD group (Figure 1). We conducted another repeated mixed model as a *post hoc* analysis to control further the effect of the history of MDE in the remaining of 167 persons after excluding all 8 cases of minDE with MDE history and 6 cases of MDD with the history. In the model, which considered the same confounders as the first model except for excluding the history of MDE, BDNF levels decreased more over time in the MDD group without the history as compared with the control group or minDD group (Figure 2).

## Discussion

Using incident cases and controls from a cohort in a 4-year observation, we found that serum BDNF levels at baseline were not lower in groups of MDD, minDD or minDE with MDE history than in controls. In contrast, decreases in serum BDNF levels from baseline to follow-up were greater in the MDD and the minDE with MDE history groups than in the control group. Furthermore, the decreases in BDNF levels from baseline to follow-up were greater in the MDD group and the minDE with MDE history group than in the minDD group.

To the best of our knowledge, we believe that the present study is the first to show that serum BDNF levels before the development of MDD and other mood disorders are not low considering a history of MDE. Several cross-sectional studies suggest that BDNF was a trait marker for depression and correlated with neuroticism.^30, 31, 32, 33, 34^ In contrast, the first cohort study^36^ did not necessarily show lower BDNF levels in an incident MDD group at baseline, although the study did not eliminate the effects of MDE history. The present longitudinal study, which considered a history of MDE or excluded all cases with the history, suggests that BDNF is not a trait marker for MDD or other types of depression. Although not statistically significant, BDNF levels at baseline seemed higher in the mood disorders groups than in the control group. This difference might correspond to increased BDNF being depressogenic in some animal studies.^29, 50, 51^

Instead of indicating that the serum BDNF level is a trait marker, our finding that its baseline-to-follow-up decrease was significantly greater in cases of MDD as compared with controls, even after controlling the effects of a history of MDE, supports the notion that it is a state marker of MDD. This notion has been suggested by several cross-sectional and cohort studies,^33, 35, 36^ which did not consider the effect of a history of MDE. Duman *et al.*,^18, 19^ however, has postulated that stress decreases BDNF in patients with MDD. Because the mean time of 28 months between baseline and the developments of MDD in the present study might be long enough for stress to decrease serum BDNF levels, the level might have decreased before MDD developed. In contrast, Bus *et al.*^36^ hypothesized that the decrease in BDNF occurs some time after the onset of depression, as their cohort study showed the baseline-to-follow-up decrease in chronic MDD but not in incident MDD. Although our study showed the decrease in incident MDD, frequent measurements of serum BDNF with short interval from baseline through follow-up, unlike the baseline-to-follow-up decrease, would be required to test the hypothesis.

Our finding that changes in serum BDNF levels from baseline to follow-up differed among the four groups is suggestive of the pathophysiologic relationships of various subcategories of mood disorders and of their classification. That the change in BDNF in the minDD group was smaller than that in the MDD group and did not differ from that in the control group suggests that the pathophysiology of minDD differs from that of MDD. In contrast, the changes in BDNF in both the MDD group and the minDE with MDE history group were greater than those in the minDD group and the control group. Furthermore, changes in BDNF levels in the MDD group and minDE with MDE history group closely mirrored one another. These findings suggest that MDD is pathophysiologically similar to minDE with MDE history. There have been arguments about whether depression that does not fulfill the criteria of MDD is a milder form of MDD or is a different category of mood disorders.^52^ The results of the present study suggest that minDD, a proposed category in DSM-IV, is a different category from MDD and that minDE with MDE history is a milder form of MDD. Differential diagnosis would be useful for selecting treatment between MDD and minDD and between phenotypically similar depressions, minDE with MDE history and minDD.^53^

The results of the present study are compelling for several reasons in addition to the use of preserved sera from a large number of subjects and a long observational period. First, the case–control study in a cohort is less likely to have recall bias than a standard case–control study. Second, obtaining numerous variables with multidisciplinary surveys allowed the study to consider the effects of confounders for any association between depression and serum BDNF.^44, 45, 46, 47, 48^ Third, we could obtain cases and controls from the same cohort. As our controls had been shown to be a representative sample from participants in the 2012 follow-up survey,^54^ the controls seem less susceptible to selection bias than controls in previous studies on serum BDNF, which were not necessarily selected from a population from which cases were.

A limitation of the present study was that the cohort providing the cases had low participation rates from the baseline survey to the follow-up surveys; hence, only the less severe cases were included in the analyses. This selection bias might also contribute to the small number of cases with a history of MDE and made the study mostly about the late onset of MDD. Furthermore, the only subjects were old women. These limitations made the results difficult to generalize. Another issue is recall bias, which potentially occurs in psychiatric surveys depending on interviews to identify previous episodes, even if the SCID, which improves the validity and reliability of diagnoses, was used. Our old subjects might not have recalled MDE from when they were younger. Although the likelihood of recall bias appears similar in cases and controls, the failure of recall in the minDD group directly affects the number of cases of minDE with MDE history. If some persons in the minDD group had actually had a history of MDE and then been transferred to the minDE with MDE history group, the serum BDNF levels during follow-up in this group would likely have increased. Therefore, the decrease in BDNF levels in this group might have been affected. We should also consider the possibility that the cases from the old cohort might include mild cognitive impairment or even dementia and, therefore, that their BDNF levels became lower,^55, 56, 57^ while we excluded persons with a low Mini-Mental State of Examination score from the analyses.

The neurotrophin hypothesis, which postulates that BDNF is involved in the development of MDD, has been supported by the low BDNF levels found in postmortem brain studies and serum studies.^2, 3, 4^ Serum studies are further expected to determine whether low BDNF levels are a state or a trait marker.^19^ Our epidemiologic approaches show that the serum BDNF level is not a trait marker of MDD in old women. Instead, the longitudinal decrease of BDNF levels in our data suggests that the serum BDNF level is a state marker of MDD, leaving the possibility that BDNF decreases several months before MDD develops. Furthermore, the different patterns of changes in serum BDNF levels among the diagnostic groups suggest that MDD has a pathophysiologic relation, not to minDD, but probably to minDE with a MDE history.

## Figures and Tables

**Table 1:** Number of participants and incident cases of mood disorders identified at psychiatrists' assessments in 2010 and 2012 follow-up surveys

	*2010*	*2012*
Participants in main body of follow-up surveys	729	570
DSBC ⩾2	108	100
DSBC ⩽1	621	470
		
Participants in psychiatrists' assessment	84	189
From all with DSBC ⩾2	44	67
A convenient sample with DSBC ⩽1	40	
From a consecutive sample of 163 with DSBC ⩽1		122
		
Incident cases of mood disorders	22	58
Major depressive disorder	8	20
Current	2	8
Partial remission	2	4
Full remission	4	6
Under treatment	0	2
Minor depressive disorder^a^	14	25
Current	13	24
Full remission	1	1
Minor depressive episode,^b^ current	0	9
With history of major depressive episode	0	8
With history of dysthymic disorder	0	1
Dysthymic disorder, current	0	2
Double depression, current	0	1
Bipolar II disorder	0	1
Non-depressed controls		106

Abbreviation: DSBC, Depression Scale Basic Checklist.

a Minor depressive disorder was diagnosed according to the criteria sets for further study in *Diagnostic and Statistical Manual of Mental Disorders*, fourth edition (DSM-IV).^41^

b Minor depressive episode was defined as depression that meets the criteria for minor depressive disorder except that there had never been a major depressive episode or a dysthymic disorder.

**Table 2:** Characteristics at follow-up by the groups of mood disorders developed between 2008 and 2012 and the control group

	*Controls (*n=*106)*	*Major depressive disorder (*n=*28)*	*Minor depressive disorder*^a^ *(*n=*39)*	*Minor depressive episode*^b^ *with history of MDE (*n=*8)*	P-*value*
Age at psychiatrists' assessments, years	81.8 (2.3)	80.5 (2.4)	81.5 (2.6)	81.6 (3.2)	0.066
MMSE at psychiatrists' assessments, points	27.8 (2.0)	27.9 (1.9)	28.3 (2.1)^c^	27.3 (2.0)	0.457
Onset age of current episode of depression, years		79.5 (2.5)	80.7 (2.7)^c^	80.6 (3.2)	0.208
Duration of illness,^d^ months		11.3 (10.8)	11.1 (10.3)^c^	11.1 (4.6)	0.998
HAMD score,^d^ points		15.7 (3.8)	9.3 (1.8)	10.3 (1.7)	0.000
Antidpressant use during follow-up period, %	—	7.1^e^	—	—	
Having history of major depressive episodes, %		21.4		100.0	
Antidepressant use in the previous MDE, %		3.6		—	
Living alone, %	41.5	28.6	38.5	50.0	0.580
Smoker, %	5.7	—	—	0.222	
Drinking alcohol 5 days or more per week, %	9.4	3.6	2.6	—	0.337
Sports or exercise activity with 1 day or less per week, %	74.5	67.9	87.2	37.5	0.021
Body mass index, kg/m^2^	22.6 (4.0)	21.3 (3.1)	21.5 (3.2)	23.2 (3.0)	0.165
History of heart diseases, %	17.0	28.6	20.5	28.6	0.529
Time difference between baseline survey and onset of current episode, months	48.0 (0.0)^f^	27.8 (13.0)	29.1 (13.8)^c^	37.8 (4.5)	0.000
Blood drawing in the afternoon, %	50.9	53.6	51.3	87.5	0.256
Fasting at blood drawing, %	1.9	—	2.6	—	0.840
Storage duration of sera,^d^ months	4.0 (0.0)	11.4 (15.6)	17.0 (17.9)	4.0 (0.0)	0.000

Abbreviations: HAMD, GRID–Hamilton Rating Scale for Depression; MDE, major depressive episode; MMSE, Mini-Mental State Examination.

Mean values (s.d.) were shown as long as % was not indicated in the first column.

a Minor depressive disorder was diagnosed according to the criteria sets for further study in *Diagnostic and Statistical Manual of Mental Disorders*, fourth edition (DSM-IV).^41^

b Minor depressive episode was defined as depression that meets the criteria for minor depressive disorder except that there had never been a major depressive episode or a dysthymic disorder.

c One missing value.

d Duration of illness, time period between the beginning of the latest episode and the follow-up survey; storage duration of sera, time period between blood drawing and brain-derived neurotrophic factor assay. Values for these items were shown for 10 persons with current episode of major depressive disorder, 37 persons with current episode of minor depressive disorder and 8 persons with current episode of minor depressive episode with MDE history.

e They were the same persons who were identified as being under treatment at follow-up.

f Time of the 2012 follow-up were used instead of onset time of onset of current episode.

**Table 3:** Serum BDNF levels and characteristics at baseline survey by the groups of mood disorders developed between 2008 and 2012 and the control group

	*Controls (*n=*106)*	*Major depressive disorder (*n=*28)*	*Minor depressive disorder*^a^ (n=*39)*	*Minor depressive episode*^b^ *with history of major depressive episode (*n=*8)*	P-*value*
Serum BDNF, ng ml^−1^	12.6 (3.6)	13.9 (3.1)^c^	13.6 (2.6)	14.5 (3.2)	0.090
Age at baseline, years	77.9 (2.3)	77.0 (2.2)	78.3 (2.4)	77.6 (3.2)	0.209
Educational level at middle school or less, %	22.6	25.0	25.6	37.5	0.811
Living alone, %	34.0	21.4	33.3	33.3	0.644
Smoker, %	6.6	3.6	2.6	0.0	0.643
Drinking alcohol 5 days or more per week, %	8.5	17.9	5.1	12.5	0.336
Sports or exercise activity with 1 day or less per week, %	66.0	71.4	82.1	62.5	0.291
Body mass index, kg/m^2^	22.8 (3.6)	22.2 (2.6)	22.1 (2.8)	22.3 (3.0)	0.559
History of heart diseases, %	16.0	21.4	28.2	28.6	0.386
Prescribed steroid, %	—	—	2.6	—	0.300
Blood drawing in the afternoon, %	31.1	32.1	48.7	62.5	0.094
Fasting at blood drawing, %	0.9	—	—	—	0.868
Storage duration of sera, months	64.4 (0.5)	65.0 (0.6)	64.7 (0.5)	64.8 (0.7)	0.000

Abbreviation: BDNF, brain-derived neurotrophic factor.

Mean values (s.d.) were shown as long as % was not indicated in the first column. Storage duration of sera, time period between blood drawing and BDNF assay.

a Minor depressive disorder was diagnosed according to the criteria sets for further study in *Diagnostic and Statistical Manual of Mental Disorders*, fourth edition (DSM-IV).^41^

b Minor depressive episode was defined as depression that meets the criteria for minor depressive disorder except that there had never been a major depressive episode or a dysthymic disorder.

c One missing value.
