# Supplementary material for: Serum BDNF levels before and after the development of mood disorders: a case–control study in a population cohort
Source: Transl Psychiatry. 2016 Apr 12;6(4):e782–. doi: 10.1038/tp.2016.47 (PMC4872405; doi:10.1038/tp.2016.47)
Supplement: Supplementary Figure 1 [file tp201647x1.pdf]

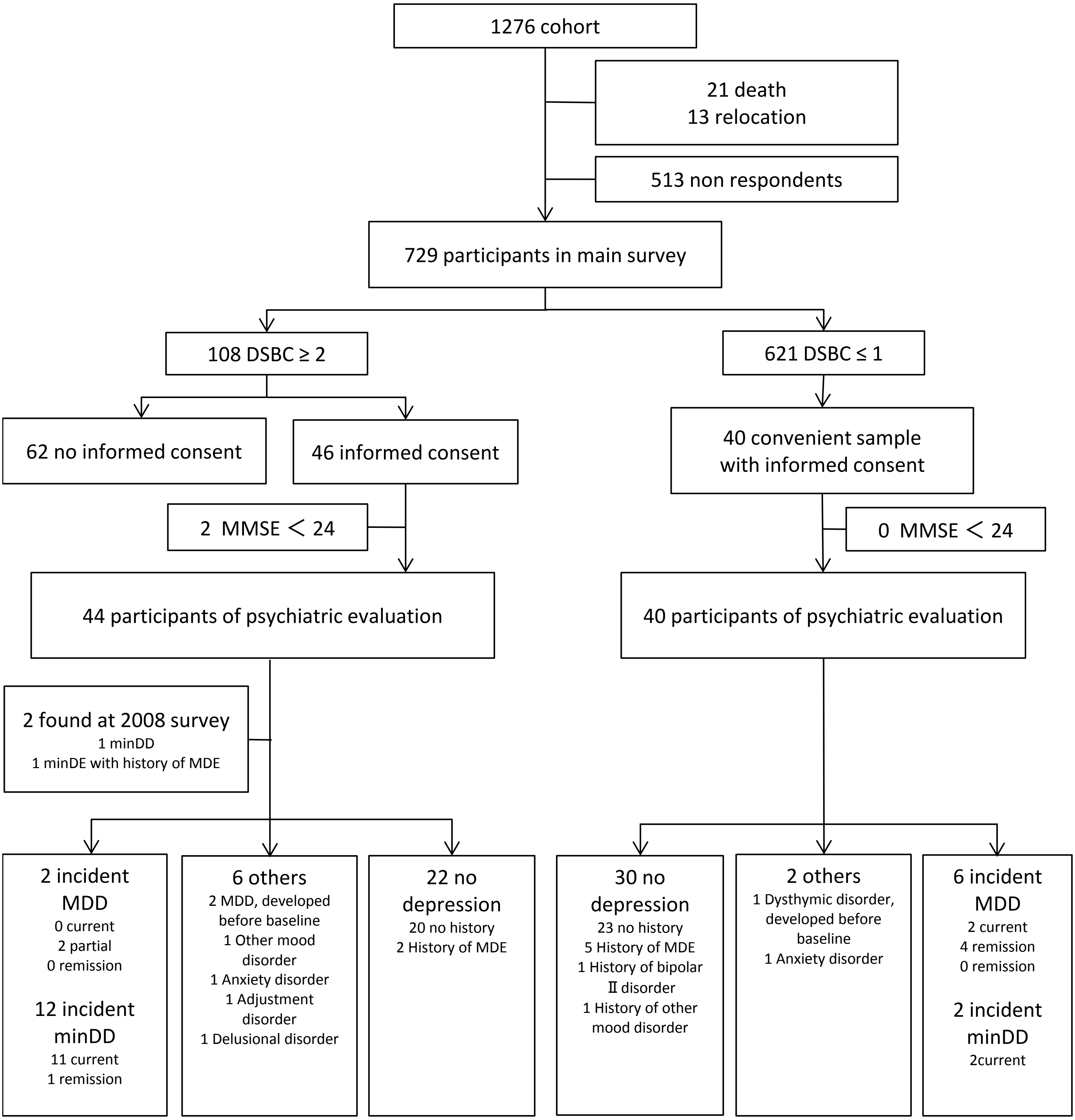

Supplementary Figure 1. Flow chart from the cohort to cases and controls at the 2010 survey.

DSBC, Depression Scale Basic Checklist; MMSE, Mini–Mental State Examination; MDD, Major Depressive Disorder; MinDD, Minor Depressive Disorder; MDE, Major Depressive Episod; minDE, minor Depressive Episode.
