# Supplementary material for: Serum BDNF levels before and after the development of mood disorders: a case–control study in a population cohort
Source: Transl Psychiatry. 2016 Apr 12;6(4):e782–. doi: 10.1038/tp.2016.47 (PMC4872405; doi:10.1038/tp.2016.47)
Supplement: Supplementary Figure 2 [file tp201647x2.pdf]

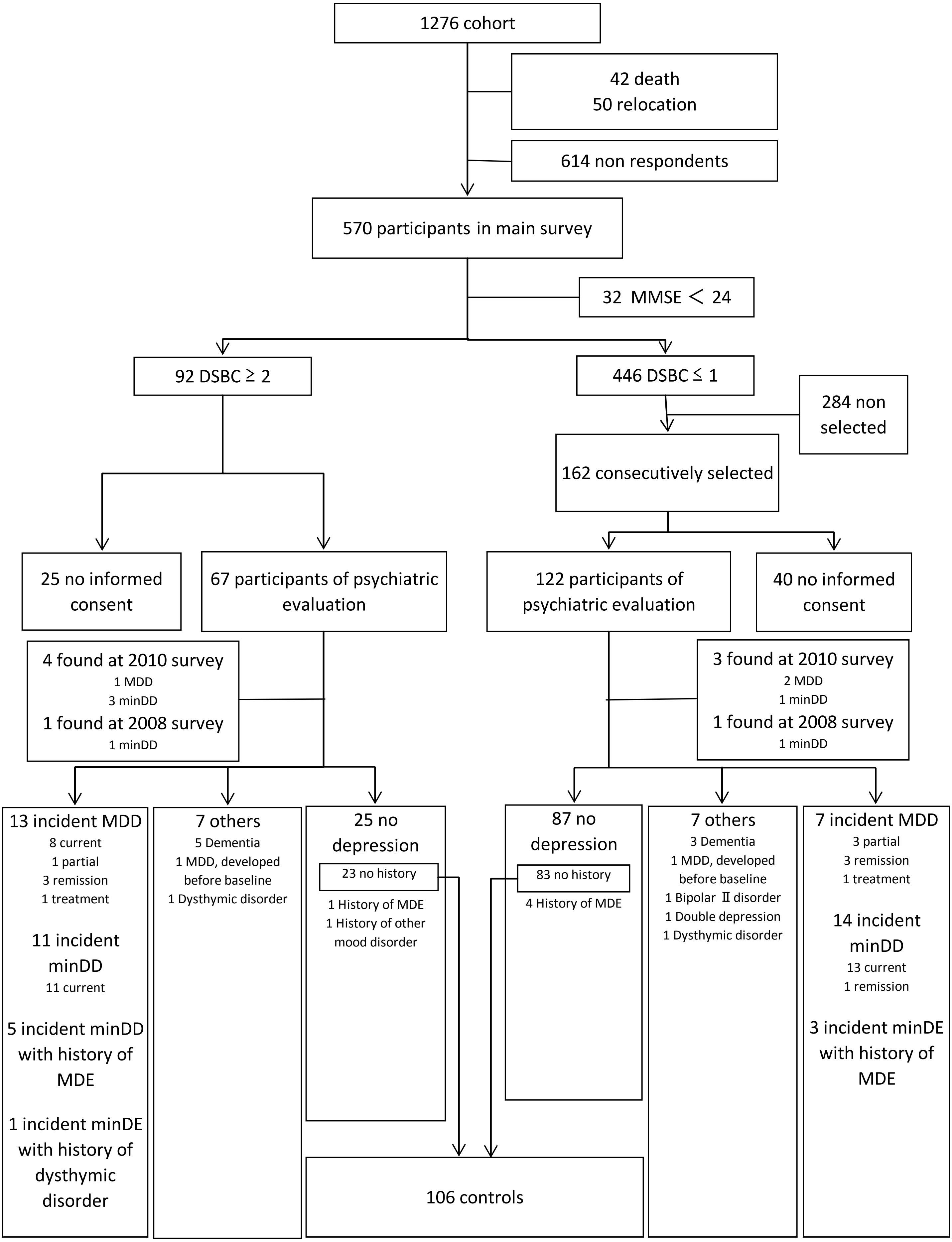

Supplementary Figure 2. Flow chart from the cohort to cases and controls at the 2012 survey.

MMSE, Mini-Mental State Examination; DSBC, Depression Scale Basic Checklist; MDD, Major Depressive Disorder; MinDD, Minor Depressive Disorder; MDE, Major Depressive Episode; minDE, minor Depressive Episode.
